# Probing Molecular Chirality via Linear Dichroism of Molecule‐Loaded Anisotropic Plasmonic Slots

**DOI:** 10.1002/nap2.70289

**Published:** 2026-09-04

**Authors:** EunSu Jeon, YoonYeong Ko, Seungwoo Choi, SeokJae Yoo

**Affiliations:** ^1^ Department of Physics Inha University Incheon Republic of Korea; ^2^ Institute of Quantum Science, Inha University Incheon Republic of Korea; ^3^ Program in Semiconductor and Device, Research and Education on Next‐Generation Semiconductor Materials and Devices for Chiplet Technology Inha University Incheon Republic of Korea

**Keywords:** chiral sensing, circular dichroism, linear dichroism, surface lattice resonance, surface plasmons

## Abstract

Conventional chiroptical spectroscopy relies on circularly polarized light (CPL), yet inherent retardance errors in CPL generation compromise signal fidelity and introduce artifacts from crosstalk with linear dichroism and birefringence. Here, we theoretically propose Chiroptical sensing via Linear dichroism of Anisotropic Plasmonic Slots (CLAPS), a CPL‐free chiroptical sensing scheme that employs two linearly polarized light (LPL) beams tilted with respect to the orientation axis of a plasmonic slot array filled with chiral analytes. Molecular optical activity perturbs the surface lattice resonance (SLR) of the array, producing a measurable reflectance difference between the two tilted LPL beams. Using an anisotropic chiral reflection model and numerical simulations, we reveal that the weak cross‐polarized reflection carrying chirality information is modulated by the strong co‐polarized response of the array. CLAPS circumvents the need for high‐purity CPL, offering a practically accessible route to trace‐level chiral molecule detection.

## Graphical abstract

We propose an optical detection strategy for chiral molecules, termed Chiroptical sensing via Linear dichroism of Anisotropic Plasmonic Slots (CLAPS). Chiral molecules are embedded within anisotropic plasmonic slot arrays, and their handedness is determined from the plasmon‐enhanced differential reflectance measured under two linearly polarized probe light excitations, oppositely tilted to the slot axis.

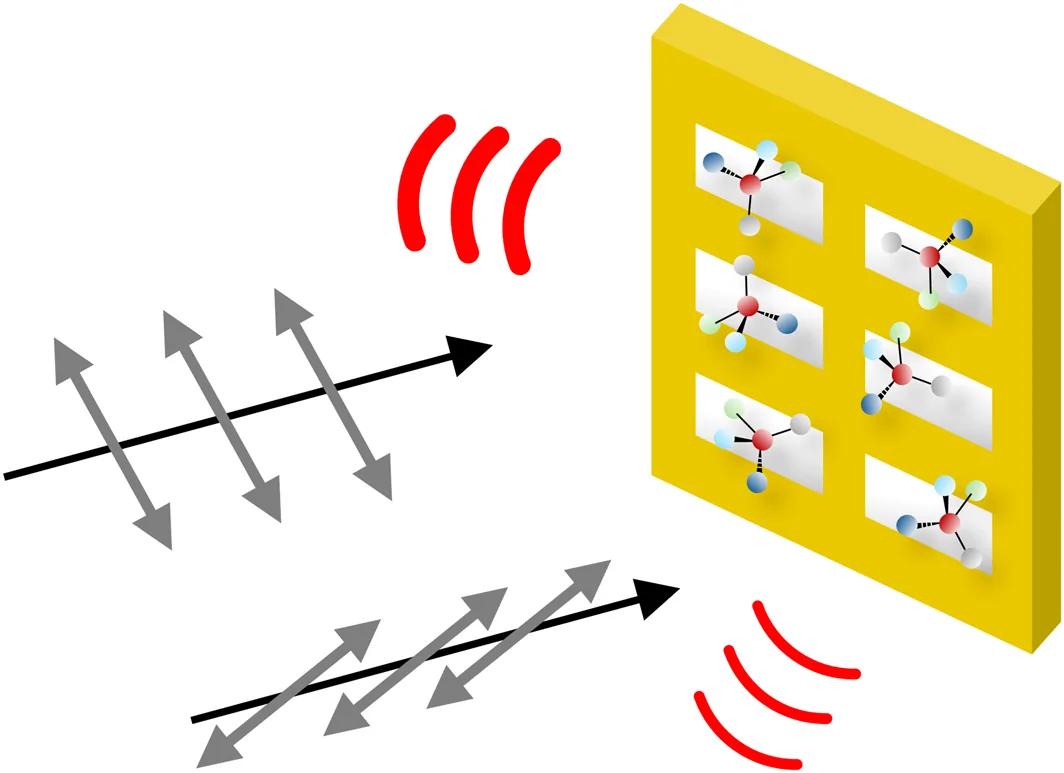

## 1 Introduction

Despite remarkable advances in optical spectroscopy, the sensitive optical detection of chiral molecules at trace concentrations continues to elude both academia and industry. Conventional chiroptical techniques, such as circular dichroism (CD) spectroscopy, typically employ circularly polarized light (CPL), to which chiral molecules respond more selectively than to any other polarization state. Even within nanophotonic frameworks to enhance molecular chiroptical signals [1, 2, 3, 4, 5, 6, 7, 8, 9, 10, 11, 12, 13, 14], CPL excitation remains a prerequisite for generating circularly polarized near‐fields at the molecular scale. However, CPL‐based optical methods present several notable limitations [15, 16]; CPL generation relies on retarders, whose inevitable retardance errors place an upper limit on CPL purity. The finite purity of CPL, in principle, introduces signal artifacts arising from crosstalk with molecular linear dichroism (LD) and birefringence. In particular, the direct subtraction method, which measures the difference in absorption between two opposite‐handed CPL beams, is highly susceptible to misalignment of optical components such as polarizers and retarders. On the other hand, while optical rotatory dispersion (ORD) is also a conventionally accessible route to probe molecular chirality, nanophotonic frameworks to enhance ORD signal is still lacking. While helical dichroism has been explored as an alternative experimental scheme for chiral sensing beyond CD and ORD [17, 18, 19, 20, 21], it remains limited by the purity of vortex generation and inevitably requires sophisticated optical instrumentation. One may then ask: is CPL truly indispensable, or can chiral molecular information instead be extracted through nanophotonic frameworks based on linearly polarized light?

A few early studies have hinted that linearly polarized light (LPL) may be used for nanophotonic chiral molecule sensing [22, 23, 24, 25, 26, 27]. However, these studies focused narrowly on nanophotonic CD enhancement via the locally confined optical helicity density of the electromagnetic field under LPL excitation. It was subsequently shown, however, that locally enhanced optical helicity density and the consequent nanophotonic CD enhancement provided was insufficient [28, 29]. Independently of these efforts, it has also been suggested that linearly polarized illumination with an achiral anisotropic metasurface can be used for chiral sensing [30]. Nevertheless, this approach still requires sophisticated polarimetry to measure both the optical rotation and ellipticity. Therefore, an experimentally feasible LPL‐based chiroptical sensing scheme has yet to be demonstrated.

Here, we propose Chiroptical sensing via Linear dichroism of Anisotropic Plasmonic Slots (CLAPS). In CLAPS, chiral analytes fill the plasmonic slots, and two LPL beams tilted relative to the slot orientation axis are incident on the array. The molecular optical activity introduces a chiral perturbation to the surface lattice resonance (SLR) of the plasmonic slot array, causing the reflected intensities of the two tilted LPLs to differ. In this work, we elucidate the mechanism of CLAPS using an anisotropic chiral reflection model and numerical simulations, revealing that the weak cross‐polarized reflection, which carries molecular chirality information, is modulated by the strong co‐polarized reflection of the plasmonic slot array. While conventional chiroptical spectroscopy such as CD suffers from technical challenges in generating high‐purity CPL, CLAPS circumvents this requirement by employing two tilted LPLs, offering a simpler and more accessible route to chiroptical spectroscopy.

## 2 Results

### 2.1 Chiroptical Sensing Using Linear Dichroism

The LD spectroscopy proposed here employs a pair of normally incident LPL beams with tilt angles ±θ with respect to the orientation axis of the chiral molecule‐loaded plasmonic slot array as shown in Figure 1a. To investigate the mechanism of CLAPS, we calculate the chiroptical response of a gold slot array with length, width, depth, and periodicity of 150 nm, 50 nm, 50 nm, and 500 nm using the finite element method (FEM). The dimensions of the slot array are the same throughout this letter. The molecules loaded in the slots are characterized by the refractive index (n=1.33) and the chirality parameter *κ*, while the constitutive relations, $\tilde{D}=\varepsilon \tilde{E}+i\kappa \tilde{H}/c$ and $\tilde{B}=\mu \tilde{H}-i\kappa \tilde{E}/c$, describe their chiroptical response.

**FIGURE 1:**
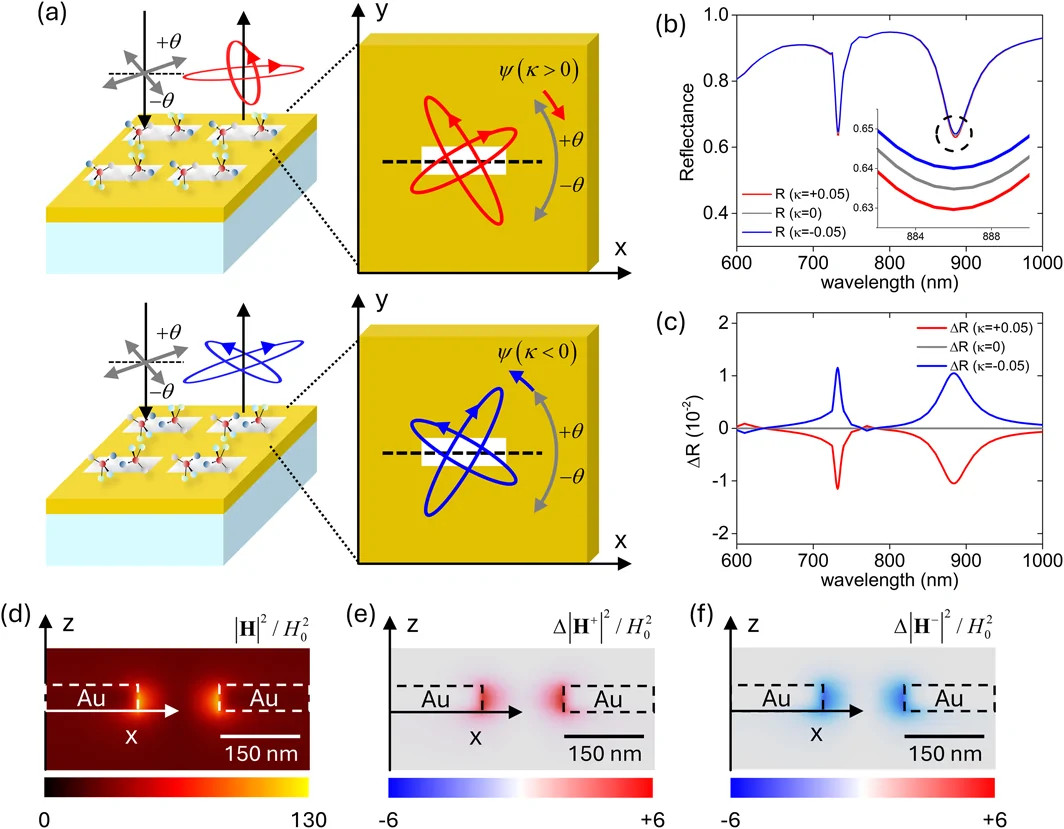
(a) Chiroptical sensing via linear dichroism of anisotropic plasmonic slots (CLAPS). The gray arrows in the inset depict a pair of linearly polarized light (LPL) beams used for linear dichroism spectroscopy with the tilt angle ± *θ* to the orientation axis of the slot. The polarization of the reflected light acquires optical rotation *ψ* by chiral molecules inside the slot. Red, blue, and gray arrows in the inset indicate optical rotation by left‐ (the chirality parameter *κ* > 0), right‐handed chiral molecules (*κ* < 0), and racemic mixture (*κ* = 0), respectively. (b) Calculated reflectance spectra of the chiral molecule‐loaded plasmonic slot arrays. (c) Differential reflectance is given by Δ*R* = *R* ^+^ − *R* ^−^, where *R* ^±^ refers to the reflectance of linearly polarized lights with polarization tilt angles ± *θ*. (d) Magnetic field intensity enhancement (|**H**|^2^/*H*_0_^2^) inside the racemic mixture‐loaded slot (κ=0). The difference in the magnetic field enhancement (Δ|**H** ^±^|^2^/*H*_0_^2^ = (|**H** ^±^|^2^−|**H** ^0^|^2^)/*H*_0_^2^) inside the chiral molecule‐loaded slot (*κ* = 0.05), where **H** ^±^ denotes the magnetic field upon LPL excitations with tilt angles (e) +*θ* and (f) −*θ*.

In the absence of chiral molecules, the reflectance *R* of the plasmonic slot array is the same for two LPLs with tilt angles ±θ because of symmetry (gray lines in Figure 1b,c). When chiral molecules are loaded inside the slots, the incident LPLs gain optical rotation *ψ* due to molecular handedness (i.e. signs of *κ*), becoming elliptically polarized. The inherently weak molecular optical rotation is modulated by strong LD of the plasmonic slot array, and then the modulated molecular optical rotation can be probed by asymmetric reflectance for two tilted LPLs (blue and red lines in Δ*R*; Figure 1b,c). In Figure 1c, the reflectance difference Δ*R* is on the order of 10^−2^ for a chiral analyte with *κ* = 0.05 confined solely within the slot with a volume of 50 × 50 × 150 nm^3^. Due to the periodicity *p*_*x*,*y*_ = 500 nm, this molecule‐filled slot volume corresponds to an effective chiral film of thickness *L* = 1.5 nm with the same *κ* and molecular volume, which yields an ORD signal of 2π*Lκ*/λ ∼ 30 mdeg. Detecting this signal via conventional polarimetry would require resolving an intensity difference of 1 − cos^2^(ORD) ≈ 10^−7^. In contrast, CLAPS yields Δ*R* ∼ 10^−2^ at the same *κ*, representing an enhancement of approximately five orders of magnitude over conventional polarimetry, as shown in Figure 1c. Figure 1d illustrates the strong optical funneling arising from SLR, mediated by the diffraction of surface plasmon polaritons on the metallic slot arrays. The resulting local magnetic field enhancement (|**H**|^2^/*H*_0_^2^) is consistent with the Babinet's principle [31]. When chiral molecules are loaded within the slots, the local magnetic fields **H** differs in the vicinity of the slots under the two LPL excitations (Figure 1e,f), revealing that the amplified chiral molecular optical rotation arises from SLR‐coupled optical funneling.

### 2.2 Anisotropic Chiral Reflection

We emphasize that chiral sensing by asymmetric reflection is clearly distinct from CD spectroscopy; isotropic chiral molecules cannot exhibit circular dichroic reflection because chirality preserves time‐reversal symmetry. The same holds for ORD spectroscopy: the optical rotation angle is recovered upon reflection, and thus cannot be probed via reflectance measurements. Therefore, conventional and nanophotonic CD and ORD spectroscopy are usually based on a transmission setup. On the other hand, as we have found in Figure 1b,c, CLAPS probes molecular chirality using a reflection setup. Here, we interpret chirality‐modulation of plasmonic LD as anisotropic chirality. The Jones reflection matrix of anisotropic medium with isotropic chirality is written as

(1) $$\left(\begin{array}{cc} r_{xx} & r_{xy}\\ r_{yx} & r_{yy} \end{array}\right)=\left(\begin{array}{cc} \frac{1-n_{x}}{1+n_{x}} & -2i\frac{n_{x}-n_{y}}{\left(1+n_{x}\right)\left(1+n_{y}\right)\left(n_{x}+n_{y}\right)}\kappa \\ -2i\frac{n_{x}-n_{y}}{\left(1+n_{x}\right)\left(1+n_{y}\right)\left(n_{x}+n_{y}\right)}\kappa & \frac{1-n_{y}}{1+n_{y}} \end{array}\right),$$

where *n*_*x*_ and *n*_*y*_ are effective refractive indices (See “Effective medium model for dielectric anisotropy and chiral isotropy” in Supporting Information S1). This yields the intensity of the reflected light as

(2) $$I\left(\theta \right)=\left({\left|r_{xx}\right|}^{2}+{\left|r_{yx}\right|}^{2}\right)\cos^{2}\;\theta +\left({\left|r_{yy}\right|}^{2}+{\left|r_{xy}\right|}^{2}\right)\sin^{2}\;\theta +\left[\text{Re}\left(r_{xx}^{*}r_{xy}+r_{yy}^{*}r_{yx}\right)\right]\sin\;2\theta .$$

Then, the difference in reflection, ΔI≡I(θ)−I(−θ)=2ψ(κ)sin(2θ), where we define optical rotation as follows:

(3) $$\psi \left(\kappa \right)\;\equiv \;\text{Re}\left[r_{xx}^{*}r_{xy}\left(\kappa \right)+r_{yy}^{*}r_{yx}\left(\kappa \right)\right].$$

If molecular chirality is absent (*κ* = 0), or anisotropy disappears (*n*_*x*_ = *n*_*y*_), cross‐polarized reflection vanishes, that is, $r_{ij}$ = 0 (*i* ≠ *j*), resulting in no optical rotation, *ψ* = 0. A remarkable feature of Equation (3) is the cross‐polarized reflection *r*_*ij*_ ≈ 0 (*i* ≠ *j*) modulated by the co‐polarized reflection *r*_*ii*_ ≠ 0, which is determined by resonantly strong and anisotropic responses of the plasmonic slots. In addition, a periodic arrangement of plasmonic slots forms a surface lattice resonance (SLR), making resonance *Q* factor significantly larger. Together with structural anisotropy of rectangular slots and the SLR formation, *r*_*ii*_ is maximally enhanced and it readily couples to chiral information *r*_*ij*_.

We calculate the spectra of the optical rotation *ψ*, the co‐polarized reflectance (*R*_*ii*_ = |*r*_*ii*_|^2^), and the cross‐polarized reflectance (*R*_*ij*_ = |*r*_*ij*_|^2^), as presented in Figure 2. Optical rotation *ψ* (Figure 2b) follows the spectral feature of differential reflectance Δ*R* shown in Figure 1c, while it increases as chirality *κ* increases, that is, more chiral molecules are loaded in the slots. Due to slot array anisotropy, *R*_*xx*_ exhibits high reflectance, whereas a clear SLR feature is shown in R_*yy*_ (Figure 2c,f). These co‐polarized reflectance spectra *R*_*ii*_ are insensitive to molecular chirality because plasmonic slot geometry dominates co‐polarized reflections. Interestingly, however, cross‐polarized reflectance *R*_*ij*_ emerges as chiral molecules are loaded inside the slots (Figure 2d,e). Therefore, we conclude that emerging cross‐polarized reflection arising from chiral molecular optical rotation gives rise to reflective LD signal Δ*R*.

**FIGURE 2:**
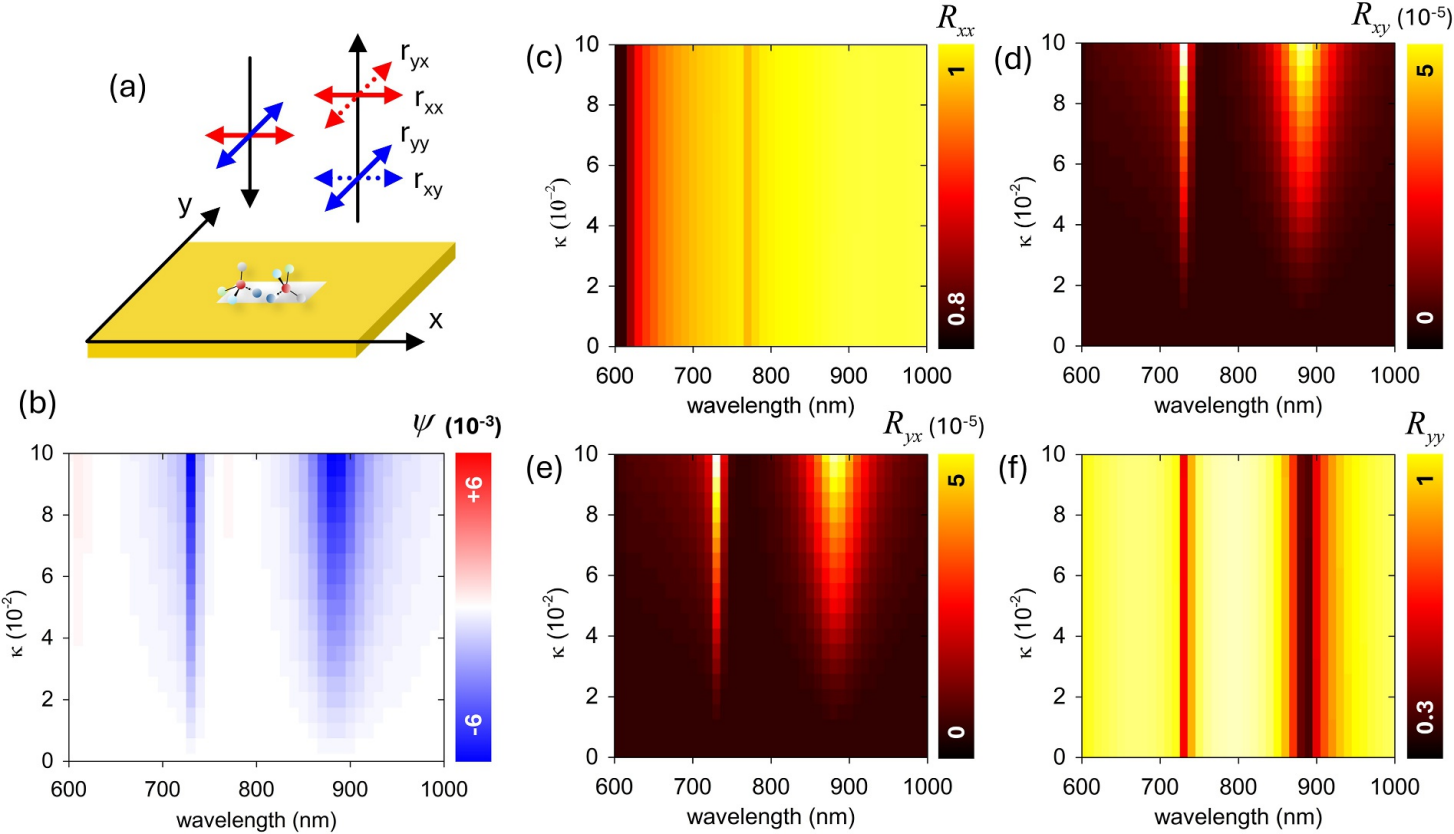
(a) The incident *x*‐ and *y*‐polarized light (red and blue arrows, respectively), and its co‐ and cross‐polarized reflection (solid and dashed arrows, respectively). (b) The spectrum of $\psi \left(\kappa \right)\;\equiv \;\text{Re}\left(r_{xx}^{*}r_{xy}+r_{yy}^{*}r_{yx}\right)$ as a function of the chirality parameter *κ*. Corresponding co‐ and cross‐polarized reflectance are calculated: (c) *R*_*xx*_, (d) *R*_*xy*_, (e) *R*_*yx*_, and (f) *R*_*yy*_.

In Figure 3, we validate the anisotropic chiral reflection model, Equations (2) and (3). We calculate Δ*R*, while we vary the tilt angle *θ* (Figure 3a,c) and chirality parameter *κ* (Figure 3b,d). Due to the anisotropy of the plasmonic slot array, Δ*R* scales with sin(2θ), in Figure 3a. Figure 3c shows a vertical cut for the wavelength *λ* = 880 nm and it agrees well with sin(2θ). From this scaling, we find that θ=45° is optimal for LD spectroscopy for chiral sensing. In Figure 3b,d, differential reflectance Δ*R* follows a linear scaling with *κ*; it is determined by the cross‐polarized reflection coefficients *r*_*ij*_, which linearly depends on *κ* according to Equations (1) and (3).

**FIGURE 3:**
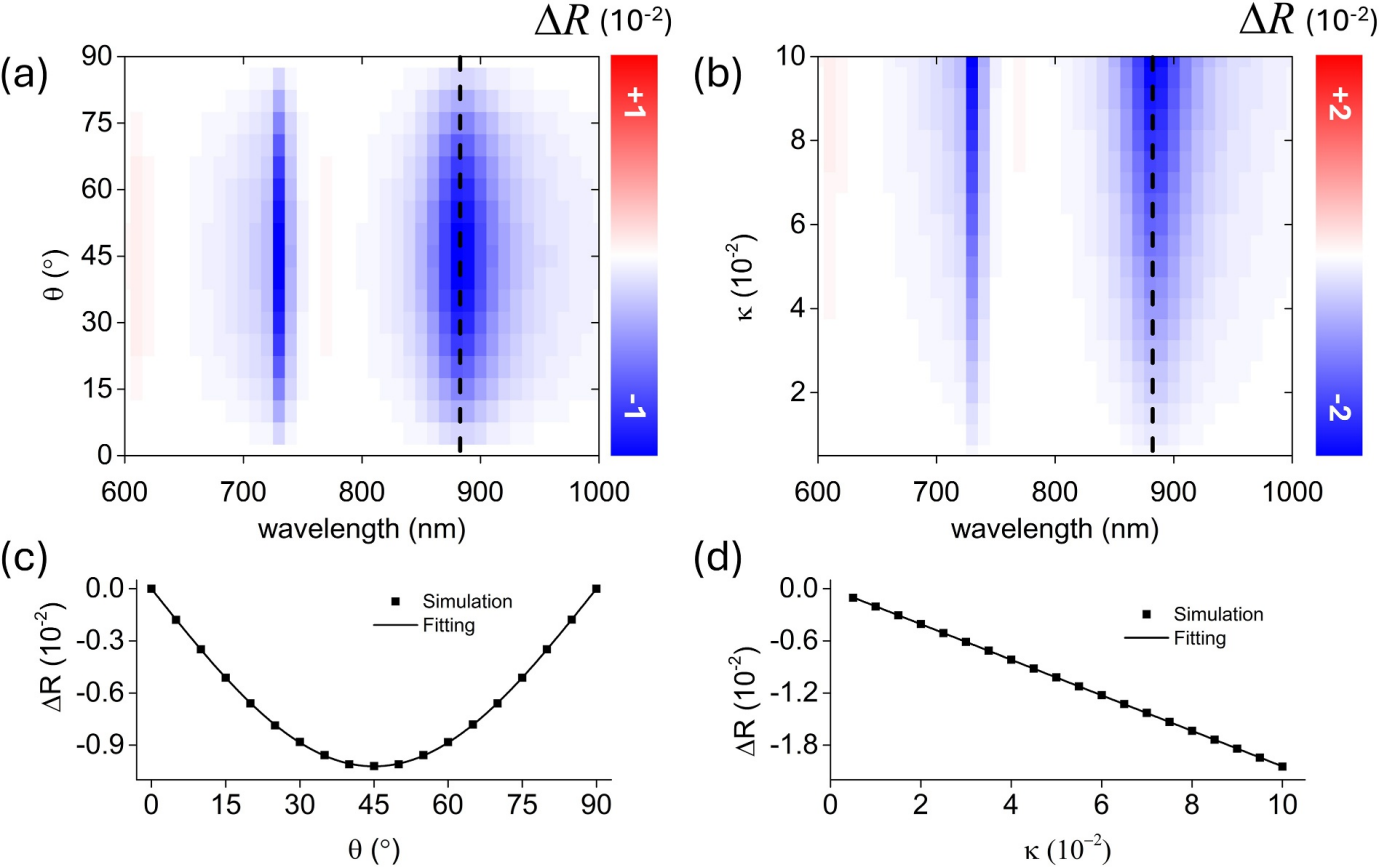
Differential reflectance spectra Δ*R* for different (a) tilt angles *θ* and (b) molecular chirality parameters *κ*. A vertical cut of Δ*R* for the (c) tilt angle and (d) molecular chirality parameter are fitted to ∼sin(2θ) and ∼*κ* at the wavelength 880 nm, respectively.

We note that Figure 3d demonstrates linear scaling for *κ* on the order of 10^−2^, but this linear scaling is expected to hold over a much smaller range of *κ* = 10^−7^−10^−4^, which is typical for chiral molecules exhibiting ORD in the visible frequency range. The limit of detection (LOD) is set by the LD measurement, that is, Δ*R*∼10^−8^−10^−7^. In our CLAPS structure, this LOD in *ΔR* can be achieved for *κ*∼10^−7^, corresponding to a molar concentration of ∼1 M [32, 33]. Given the extremely small volume of the plasmonic slot, this corresponds to attomole‐level detection of chiral molecules, surpassing the conventional LOD of CD and ORD spectroscopy. The signal‐to‐noise ratio (SNR) is proportional to $\propto \Delta I/\sqrt{I}=\Delta RI_{0}/\sqrt{RI_{0}}=\sqrt{I_{0}}\left(\Delta R/\sqrt{R}\right)$, where $I_{0}$ is the incident beam intensity. An optimal SNR can be obtained through strong plasmonic anisotropy (i.e., large Δ*R*) and a high‐*Q* resonance (i.e., small *R*) together with a strong beam intensity to minimize shot noise.

### 2.3 Comparison of LD‐Signal Between Complementary Structures

We have shown that our approach can probe chiral molecules and their handedness information using LPLs. A natural question then arises: can any anisotropic nanostructure serve this purpose? To answer this question, we compare the slot array (Figure 4a) with its complementary structure, that is, a rod array immersed in chiral molecules (Figure 4b). In Figure 4c,d, we calculate reflectance spectra of these two structures as a function of lattice periodicity. Reflectance spectra show a clear SLR feature guided by the Rayleigh anomalies (RA) of the empty lattice (solid and dashed lines in Figure 4c,d). SLR of the slot array arises from strongly coupled SPPs propagating along the air‐metal and substrate‐metal interfaces, while that of the rod array arises from diffractive optical modes formed at the interface between two dielectrics [34, 35]. Thus, a clear signature of distinctive SLR is featured by the reflectance dip and peak from slot and rod arrays, respectively [31, 36].

**FIGURE 4:**
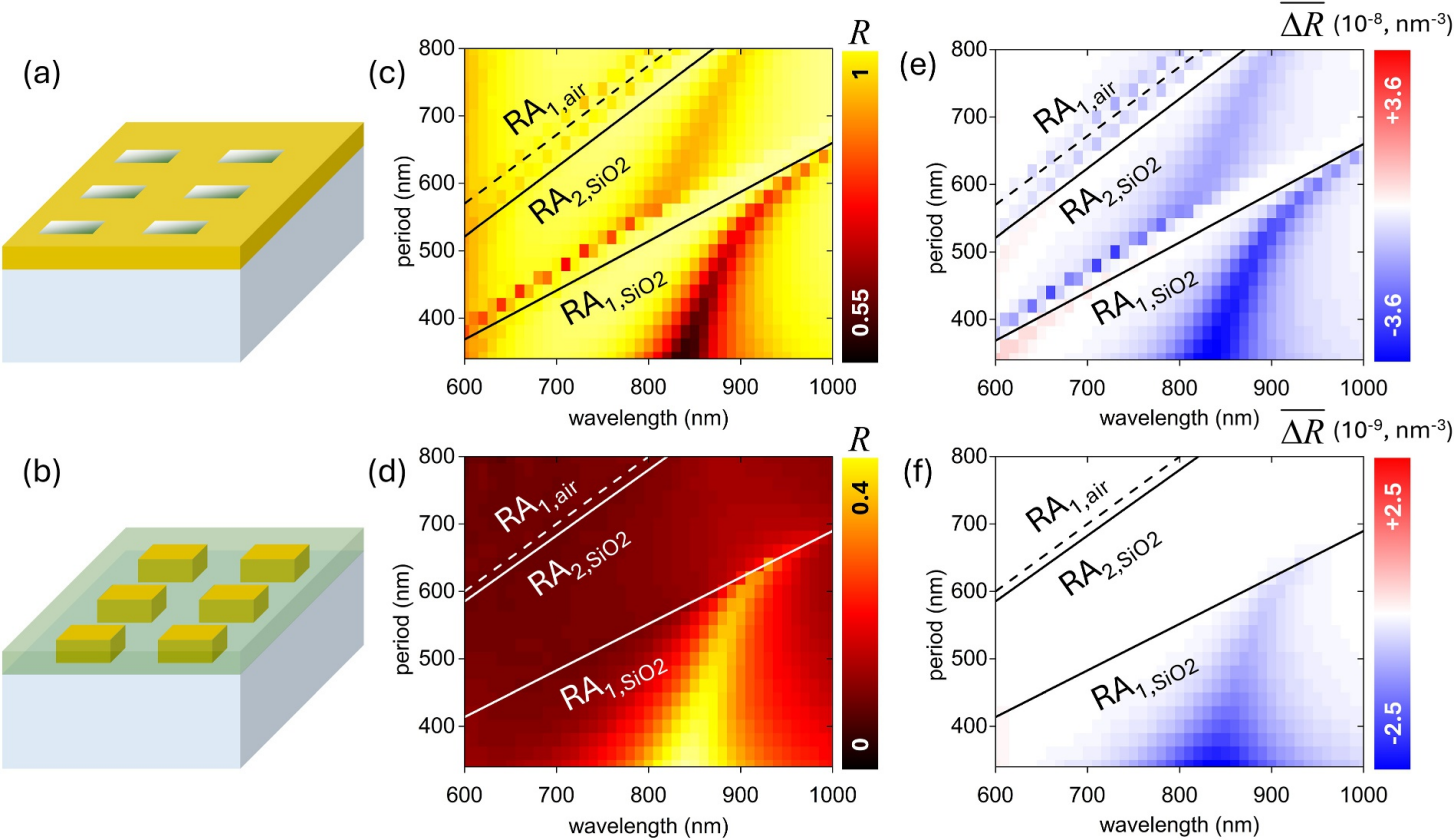
Structures of (a) the chiral molecule‐loaded slot array and its complementary structure, (b) the rod array immersed in chiral molecules. Reflectance spectra *R* of (c) the slot and (d) the rod array upon the LPL excitation with the tilt angle θ=45° (solid and dashed lines: Rayleigh anomalies; RA, the subscript denotes the RA order and the interface where RA occurs). Normalized differential reflectance, $\bar{\Delta R}\;\equiv \;\Delta R/V_{\text{mol}}$, where $V_{mol}$ is volume occupied by molecules of (e) the slot and (f) the rod array.

Since the number of molecules loaded in the slot array differs from that in the rod array, we normalize differential reflectance by molecular volume *V*_mol_ for a fair comparison: $\bar{\Delta R}\;\equiv \;\Delta R/V_{\text{mol}}$ (see Supporting Information S1: Figure S1 for the unnormalized differential reflectance). Comparing the normalized differential reflectance spectra in Figure 4e,f, we find that $\bar{\Delta R}$ of the slot array (Figure 4e) is an order of magnitude larger than that of its complementary counterpart (Figure 4f). In the slot array, the incident light is strongly confined inside the slot by the funneling effect [35, 37, 38, 39], and molecular chirality is efficiently transferred to the plasmonic anisotropy of the slots. On the other hand, the molecule‐plasmon coupling occurs mainly near the rod tips (Supporting Information S1: Figure S2). It is noteworthy that the rectangular slot array exhibits over an order of magnitude larger differential reflectance than CD of the circular hole array because the circular hole is a symmetric structure lacking strong LD to modulate molecular chirality (Supporting Information S1: Figure S3) [40, 41]. While we used comparatively large chirality parameter (*κ* = 0.05) to clearly illustrate the reflectance splitting (Figure 1b), comparative study in Supporting Information S1: Figure S3 confirms that CLAPS mechanism remains valid at a realistic chirality parameter (*κ* = 10^−4^ + 10^−6^ *i*). To summarize, the slot array is well‐suited for our LD spectroscopy‐based chiral sensing scheme because of its light funneling effect and strong plasmonic anisotropy.

### 2.4 Probing Complex Molecular Chirality Using CLAPS

We have considered chiral molecules exhibiting ORD alone (i.e., a purely real‐valued *κ*) in the visible and near‐infrared spectral range so far because CD, characterized by the imaginary part of *κ*, survives only in the ultraviolet range for most chiral molecules in nature. However, previous nanophotonic chiral sensing studies mainly focused on the nanophotonic enhancement of molecular CD; it has been suggested that the strong optical helicity density *h*, also known as the optical chirality density *C*, of the local electromagnetic fields can enhance molecular CD [42, 43]. Here, we also elucidate the effect of molecular CD on CLAPS.

In Figure 5a, we calculate the differential reflectance Δ*R* of the slot array filled with ORD‐only ($\kappa^{\prime }=0.05$), CD‐only ($\kappa^{\prime \prime }=0.05i$), and mixed ($\kappa^{\prime }+\kappa^{\prime \prime }$) molecules. While they provide Δ*R* at the same order, their spectral lineshapes are different. Interestingly, co‐ and cross‐polarized reflectance *R*_*ij*_ = |*r*_*ij*_|^2^ are the same for both cases (Figure 5b–e), but the cross‐polarized reflection is out‐of‐phase by a factor of π/2 attributed to the linear scaling behavior of *r*_*ij*_(*κ*) (Figure 5f). This gives rise to the retrieval of the complex molecular chirality by relating between ORD‐only and CD‐only molecules (Equation (4)).

(4) $$\psi \left(\kappa^{\prime \prime }\right)=\hat{\psi }\left(\kappa^{\prime }\right)\;\equiv \;\text{Im}\left[r_{xx}^{*}r_{xy}\left(\kappa^{\prime }\right)+r_{yy}^{*}r_{yx}\left(\kappa^{\prime }\right)\right].$$

**FIGURE 5:**
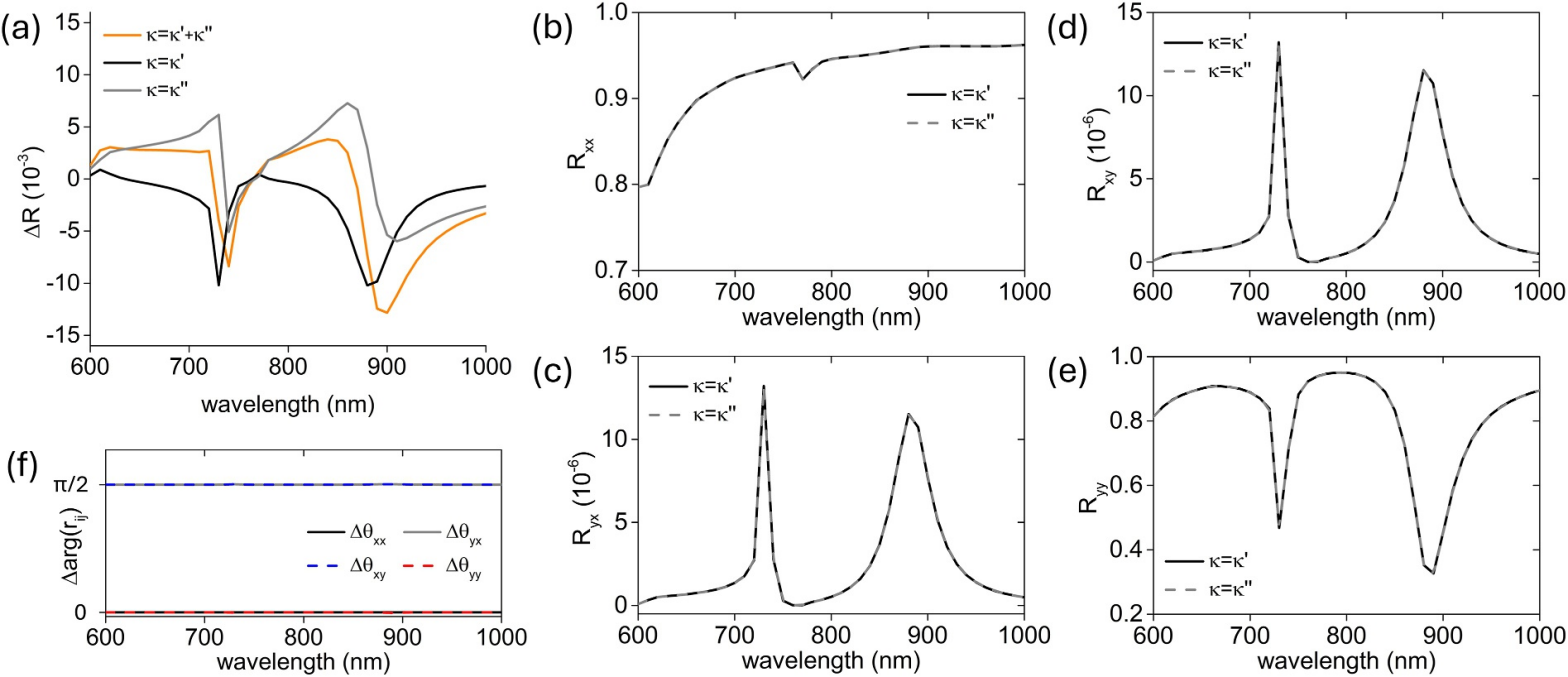
(a) Differential reflectance of the slot array filled with chiral molecules of the complex‐, purely real‐, and imaginary‐valued chirality parameters, $\kappa^{\prime }+\kappa^{\prime \prime }$, $\kappa^{\prime }=0.05$, and $\kappa^{\prime \prime }=0.05i$, respectively. Co‐ and cross‐polarized reflectance are calculated: (b) *R*_*xx*_, (c) *R*_*xy*_, (d) *R*_*yx*_, and (e) R_yy_. (f) Difference in argument of reflection coefficients, $\Delta \arg\left(r_{ij}\right)=\arg\left[r_{ij}\left(\kappa^{\prime }\right)\right]-\arg\left[r_{ij}\left(\kappa^{\prime \prime }\right)\right]$.

This explains why the spectral lineshape of Δ*R* differs according to the complex‐valued chirality parameter *κ*, that is, a combination of molecular ORD and CD. Equation (4) is further validated by numerical simulation (Supporting Information S1: Figure S4). Since *r*_*ij*_ scales linearly with *κ*, the optical rotation of analyte molecules exhibiting both ORD and CD can be decomposed into $\psi \left(\kappa^{\prime }+\kappa^{\prime \prime }\right)=\psi \left(\kappa^{\prime }\right)+\psi \left(\kappa^{\prime \prime }\right)$. This suggests that reconstruction of the complex‐valued chirality parameter spectrum, and thus extraction of stereochemical information, is possible using CLAPS. Considering the spectral lineshape is dependent on molecular ORD and CD, it is worth noting that CLAPS may enable deconvolution of molecular signals from the complicated CLAPS signals; this remains an interesting avenue for future investigation.

## 3 Conclusion

In conclusion, we propose CLAPS as a means of overcoming the key limitations of conventional chiroptical spectroscopy: the difficulty of generating high‐purity CPL and the complexity of the associated optical setup. In CLAPS, differential reflectance is measured upon the excitation of two LPLs tilted relative to the plasmonic slot axis. While differential reflectance vanishes for analytes of achiral molecules or racemic mixtures, it appears only when analytes filled in the slot are chiral. We also reveal the mechanism behind CLAPS; it exploits the coupling of chiral molecular information, encoded in the cross‐polarized reflection, to the strong co‐polarized plasmonic response. It is also noteworthy that CLAPS exploits molecular chirality transfer to nanophotonic resonance, a mechanism that has been shown to effectively amplify chiroptical signals in the previous works [1, 8, 44, 45, 46], while uniquely employing LPL to decode chiral information from the cross‐polarized reflection, circumventing the need for CPL altogether. Furthermore, because CLAPS operates on the complex molecular chirality, it offers a robust guideline for future experiments beyond current studies limited to enhancing the local helicity density [28, 29]. Our theoretical study not only substantially reduces instrumental complexity, but also opens a new avenue for chiroptical sensing that transcends the fundamental limitation of inherently weak molecular CD.

## Author Contributions

**EunSu Jeon:** conceptualization, investigation, formal analysis, writing – original draft, writing – review and editing, software, methodology, validation, visualization. **YoonYeong Ko:** investigation, software. **Seungwoo Choi:** software, investigation. **SeokJae Yoo:** conceptualization, methodology, writing – original draft, writing – review and editing, funding acquisition, investigation, supervision, formal analysis, project administration, resources, validation.

## Funding

This study was supported by the National Research Foundation of Korea (Grant Nos. RS‐2025‐16067780 and RS‐2026‐25480823) and the Korea Institute for Advancement of Technology (Grant No. RS00411221).

## Conflicts of Interest

The authors declare no conflicts of interest.

## Supporting information


Supporting Information S1

## Data Availability

The data sets generated and analyzed during this study are available from the corresponding author upon reasonable request.
