# Supplementary material for: Probing Molecular Chirality via Linear Dichroism of Molecule‐Loaded Anisotropic Plasmonic Slots
Source: Nanophotonics. 2026 Sep 4;15(17):e70289. doi: 10.1002/nap2.70289 (PMC13672156; doi:10.1002/nap2.70289)
Supplement: Supplementary file 1 [file NAP2-15-e70289-s001.docx]

**Supporting Information:**

**Probing Molecular Chirality via Linear Dichroism of Molecule-Loaded Anisotropic Plasmonic Slots**

EunSu Jeon^1,2^, YoonYeong Ko^1,2^, Seungwoo Choi^1,2^, SeokJae Yoo^1,2,3^*

^1^Department of Physics, Inha University, Incheon 22212, Republic of Korea

^2^Institute of Quantum Science, Inha University, Incheon 22212, Republic of Korea

^3^Program in Semiconductor and Device, Research and Education on Next-Generation Semiconductor Materials and Devices for Chiplet Technology, Inha University, Incheon 22212, Republic of Korea

**Simulation method**

We performed numerical simulations in the main text and the supporting information (SI) using the finite element method (FEM) provided by COMSOL Multiphysics. The permittivity of gold was taken from tabulated data [1]. The refractive index of the SiO_2_ substrate was set as 1.45. Chiroptical response of molecules was modeled by the electromagnetic constitutive relations, $\tilde{\mathbf{D}}=\varepsilon\tilde{\mathbf{E}}+i\kappa\tilde{\mathbf{H}}/c$ and $\tilde{\mathbf{B}}=\mu\tilde{\mathbf{H}}-i\kappa\tilde{\mathbf{E}}/c$, for the isotropic chiral medium [2]. We note that the chirality parameter, $\kappa=0.05$, which is larger than typical values in the visible range (10^-7^ – 10^-4^), was used in the main text to clearly demonstrate the reflectance splitting (Figure 1a). In Figure S3, however, we used $\kappa=10^{-4}+10^{-6}i$ to reflect a typical value of small chiral molecules. Periodic boundary conditions were applied to model periodically arranged slots with chiral molecule loading. To avoid the numerical artifact for the weak chiroptical response, we adopted symmetric meshing strategy in our FEM simulation [3].

In the main text, co- and cross-polarized reflectance as well as optical rotation *ψ*(*κ*) are computed using FEM simulations. They consist of co- and cross-polarized reflection coefficients, calculated by the ratio between reflected and incident electric fields, $r_{ij}=\mathbf{E}_{ref,i}/\mathbf{E}_{inc,j}$, where $\mathbf{E}_{ref,i}=\mathbf{E}_{tot,i}-\mathbf{E}_{inc,i}$.

**Effective medium model for dielectric anisotropy and chiral isotropy**

We model the anisotropic plasmonic slot array filled with isotropic chiral molecules as the effective medium with the anisotropic refractive indices *n*_x_ and *n*_y_ and the isotropic chirality parameter *κ*. Here we derive the reflection coefficient matrix used in the main text. First of all, we can obtain the wave equation for the effective medium, $\mathbf{A}\left(E_{x},E_{y}\right)^{t}=\mathbf{0}$, where the matrix **A** is given by
$\mathbf{A}=\left(\begin{matrix} k_{0}^{2}\varepsilon_{x}-k^{2}-k_{0}^{2}\kappa^{2} & -2ikk_{0}\kappa\\ 2ikk_{0}\kappa& k_{0}^{2}\varepsilon_{y}-k^{2}-k_{0}^{2}\kappa^{2} \end{matrix}\right)$

where $\varepsilon_{x}=n_{x}^{2}$ and $\varepsilon_{y}=n_{y}^{2}$. We define $\bar{\varepsilon}=\left(\varepsilon_{x}+\varepsilon_{y}\right)/2$ and $\Delta\varepsilon=\left(\varepsilon_{x}-\varepsilon_{y}\right)/2$ for the sake of simplicity. We have eigenvalues for the wave numbers *k*_1(2)_ in the medium from $\det\mathbf{A}=0$ by neglecting $O\left(\kappa^{2}\right)$ for small molecular chirality *κ*.
$\begin{matrix} k_{1\left(2\right)}^{2}=k_{0}^{2}\left[\left(\bar{\varepsilon}+\kappa^{2}\right)\pm\sqrt{\Delta\varepsilon^{2}+4\kappa^{2}\bar{\varepsilon}}\right] \\ \approx k_{0}^{2}\varepsilon_{x\left(y\right)} \end{matrix}$

The eigenvectors corresponding to *k*_1(2)_ provide the electric fields **E**^(1)^ and **E**^(2)^,
$\mathbf{E}^{\left(1\right)}=\left(\begin{matrix} 1 \\ \frac{in_{x}}{\Delta\varepsilon}\kappa\end{matrix}\right),\text{ }\mathbf{E}^{\left(2\right)}=\left(\begin{matrix} \frac{in_{y}}{\Delta\varepsilon}\kappa\\ 1 \end{matrix}\right)$

To derive reflection matrix, we use the continuity equations of electric and magnetic fields, $\mathbf{E}_{inc}+\mathbf{E}_{ref}=\mathbf{E}_{tra}$ and $\mathbf{H}_{inc}+\mathbf{H}_{ref}=\mathbf{H}_{tra}$. Since a transmitted light is expressed by linear combination of eigenvectors in anisotropic chiral medium, both continuity equations are rewritten as system .
$\begin{matrix} \mathbf{E}_{inc}+\mathbf{E}_{ref}=a_{1}\mathbf{E}_{tra}^{\left(1\right)}+a_{2}\mathbf{E}_{tra}^{\left(2\right)} \\ \mathbf{E}_{inc}-\mathbf{E}_{ref}=a_{1}\left(n_{1}\mathbf{1}+i\kappa\mathbf{J}\right)\mathbf{E}_{tra}^{\left(1\right)}+a_{2}\left(n_{2}\mathbf{1}+i\kappa\mathbf{J}\right)\mathbf{E}_{tra}^{\left(2\right)} \end{matrix}$,

where the matrix **J** is defined as,

$$\mathbf{J}\equiv\left(\begin{matrix} 0 & 1 \\ -1 & 0 \end{matrix}\right)$$

Consequently, the incident and the reflected light are simply represented to
$\begin{matrix} \mathbf{E}_{inc}=a_{1}\mathbf{P}_{1}\mathbf{E}_{tra}^{\left(1\right)}+a_{2}\mathbf{P}_{2}\mathbf{E}_{tra}^{\left(2\right)} \\ \mathbf{E}_{ref}=a_{1}\mathbf{Q}_{1}\mathbf{E}_{tra}^{\left(1\right)}+a_{2}\mathbf{Q}_{2}\mathbf{E}_{tra}^{\left(2\right)} \end{matrix}$,

where $\mathbf{P}_{1\left(2\right)}=\left[\left(1+n_{x\left(y\right)}\right)\mathbf{1}+i\kappa\mathbf{J}\right]/2$ and $\mathbf{Q}_{1\left(2\right)}=\left[\left(1-n_{x\left(y\right)}\right)\mathbf{1}-i\kappa\mathbf{J}\right]/2$.
Finally, we have the reflection coefficient matrix for the effective media, equation (1)
$\begin{matrix} \mathbf{r}=\left(\begin{matrix} \mathbf{Q}_{1}\mathbf{E}_{tra}^{\left(1\right)} & \mathbf{Q}_{2}\mathbf{E}_{tra}^{\left(2\right)} \end{matrix}\right)\left(\begin{matrix} \mathbf{P}_{1}\mathbf{E}_{tra}^{\left(1\right)} & \mathbf{Q}_{2}\mathbf{E}_{tra}^{\left(2\right)} \end{matrix}\right)^{-1} \\ =\left(\begin{matrix} \frac{1-n_{x}}{1+n_{x}} & -2i\frac{n_{x}-n_{y}}{\left(1+n_{x}\right)\left(1+n_{y}\right)\left(n_{x}+n_{y}\right)}\kappa\\ -2i\frac{n_{x}-n_{y}}{\left(1+n_{x}\right)\left(1+n_{y}\right)\left(n_{x}+n_{y}\right)}\kappa& \frac{1-n_{y}}{1+n_{y}} \end{matrix}\right) \end{matrix}$

We note that coexistence of anisotropy and molecular chirality gives rise to the nonzero cross-polarized reflection.

Figure S1 Differential reflectance of chiral molecule loaded (a) slot array and (b) rod array. In Fig. S1, chiral molecules are modeled as homogeneous chiral medium with the refractive index *n*_mol_ = 1.33 and the chirality parameter *κ* = 0.05.

Figure S2 (a) Electric field enhancement averaged over the surface of (a) the slot and (b) the rod array unit cell. Spatial profiles of the electric field enhancement, $\left|\mathbf{E}\right|^{2}/E_{0}^{2}$ in the yz-cross section of (c) the slot array unit cell and in the xz-cross section of (d) the rod array unit cell.

Figure S3 Structures of the chiral molecule-loaded (a) slot, (b) rod, and (c) circular hole array. Reflectance spectra of (d) the slot, (e) the rod, and (f) the hole array. For the slot and rod array in Fig. S3a&b, CLAPS is used to obtain differential reflectance of two linearly polarized light tilted relative to the orientation axis of slots and rods as mentioned in the main text. For the hole array, circular dichroism (CD) is used to obtain difference in absorption of two opposite circularly polarized light beams. Differential reflectance (in CLAPS) and absorptance (in CD) is normalized by the molecular volume *V*_mol_: $\overline{\Delta R}\equiv\Delta R/V_{mol}$ of (g) the slot and (h) the rod array. (i) $\overline{\Delta A}\equiv\Delta A/V_{mol}$ of the hole array. Solid and dashed lines denote the Rayleigh anomalies (RA) of the empty lattice in Fig. S3d-i. Dimensions of the slot and rod array are the same as the main text. For the hole array, radius and depth are 90 nm and 50 nm, respectively. In Fig. S3, chiral molecules are modeled as homogeneous chiral medium with the refractive index *n*_mol_ = 1.33 and the chirality parameter *κ* = 10^-4^+10^-6^*i*.

**Figure S4** For purely real (*κ*’=0.05) and imaginary (*κ*”=0.05*i*) chirality parameters, the optical rotation $\psi\left(\kappa''\right)\equiv\mathrm{Re}\left[r_{xx}^{*}r_{xy}\left(\kappa''\right)+r_{yy}^{*}r_{yx}\left(\kappa''\right)\right]$ and its conjugated counterpart $\hat{\psi}\left(\kappa'\right)\equiv\mathrm{Im}\left[r_{xx}^{*}r_{xy}\left(\kappa'\right)+r_{yy}^{*}r_{yx}\left(\kappa'\right)\right]$ are calculated using the FEM method.

[1]	P. B. Johnson and R. W. Christy, Optical Constants of the Noble Metals, Phys. Rev. B **6**, 4370 (1972).

[2]	I. V. Lindell, A. H. Sihvola, S. A. Tretyakov, and A. J. Viitanen, *Electromagnetic Waves in Chiral and Bi-Isotropic Media* (Artech House, 1994).

[3]	S. Lee, J. H. Kang, S. J. Yoo, and Q. H. Park, Robust numerical evaluation of circular dichroism from chiral medium/nanostructure coupled systems using the finite-element method, Sci Rep **8**, 1 (2018).
